# Anemia from A to zinc: Hypocupremia in the setting of gastric bypass and zinc excess

**DOI:** 10.1002/ccr3.2741

**Published:** 2020-02-26

**Authors:** Vineeth Tatineni, Julie Y. An, Matthew R. Leffew, Sameer A. Mahesh

**Affiliations:** ^1^ Department of Internal Medicine Summa Health Akron City Hospital Akron OH USA; ^2^ College of Medicine Northeast Ohio Medical University Rootstown OH USA; ^3^ Department of Pathology Summa Health Akron City Hospital Akron OH USA; ^4^ Department of Hematology/Oncology Summa Health Akron City Hospital Akron OH USA

**Keywords:** gastroenterology and hepatology, hematology, nutrition, pharmacology

## Abstract

Hypocupremia can result in a bi‐lineage deficiency of leukocytes and erythrocytes. Although commonly seen from gastrointestinal malabsorption, hypocupremia can be further exacerbated with excessive zinc intake causing increased fecal copper excretion. Dietary supplementation is prevalent in the outpatient setting and must be considered as a possible source of hematologic pathologies.

## Graphical abstract

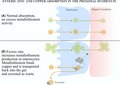

## 1 INTRODUCTION

Zinc and copper are both essential trace elements and enzymatic co‐factors in systemic regulatory pathways. Copper is involved with cellular oxidation, cross‐linking of collagen, lipid metabolism, myelin formation, and erythropoiesis. Zinc plays a crucial role in the immune system through cellular proliferation and RNA and DNA synthesis.^1^, ^2^, ^3^ Deficiencies and toxicities in copper are rare due to modern diets and the body's tight control of copper absorption and exertion.^1^, ^4^ Copper deficiency can be seen in certain incidences where there is insufficient absorption in the duodenum due to myelodysplastic syndromes, parenteral nutrition, tube feedings, gastric bypass, or zinc excess.^5^ Zinc deficiency is seen mostly from nutritional factors and is adequately treated through repletion of zinc stores.^6^ Zinc toxicity occurs, most often, via excess supplementation and can infrequently lead to cellular apoptosis.^6^ However, most adverse effects seen from zinc toxicity are secondary to zinc‐induced copper deficiency.

Both zinc and copper compete for absorption by erythrocytes in the proximal duodenum. Metallothioneins, a type of ubiquitin protein present in the proximal duodenum, can become upregulated when an excess of either element is detected and bind to and excrete the element.^6^ However, metallothioneins have a higher affinity to copper than zinc; therefore, copper excretion can be provoked by an excess of dietary zinc.^6^ In this report, we describe a case of significant anemia and neutropenia due to copper deficiency in the setting of gastric bypass compounded by a mixed clinical picture with zinc excess and note the relevance of dietary supplementation in hematological pathologies.

## 2 PATIENT PRESENTATION

A 62‐year‐old female presented to the emergency department (ED) with fatigue, intermittent chest pain, and shortness of breath for several weeks. She also had nonspecific abdominal pain, constipation, nausea, arthralgias, back pain, myalgias, neck stiffness, weakness, and lightheadedness. In the ED, she was found to be severely anemic and neutropenic, with a hemoglobin (Hgb) of 6.5 g/dL and an absolute neutrophil count (ANC) of 500 cells/μL. Platelet count was within normal limits, at 291 000 cells/μL. The patient's past medical history was significant for hypertension, arthritis, and gastric bypass surgery that was performed 14 years ago. Psychiatric history was significant for depression, past suicide attempt with acetaminophen overdose 10 years ago, and PTSD from sexual assault in her 20s. The patient was previously diagnosed with hypothyroidism and placed on levothyroxine. However, she was taken off this medication by her primary care provider about a year ago. Since then, she had been taking 50 mg of zinc twice a day, because she heard on a syndicated medical TV show that it improves thyroid function. Additional home medications include metoprolol, lisinopril, aspirin, and biotin. She denied smoking, illicit drug use, and attested to rare alcohol use. Family history was significant for autoimmune disease and hypothyroidism.

This patient's history opened her to many possible causes for anemia and neutropenia. Several weeks of nonspecific myalgias, arthralgias, and fatigue raises suspicion for an acute viral infection, while a history of gastric bypass puts one at risk for malnutritional‐related cytopenias from deficiency of dietary vitamin and minerals (eg, vitamin B12, folate, copper). Alternatively, the patient could have had an underlying, undiagnosed autoimmune condition given her family history and prior thyroid disease, and the use of zinc supplementation puts her at risk for copper deficiency‐related anemia. The patient was not on any psychotropic medications that predispose to aplastic anemia, but her prior psychiatric history and suicide attempt would prompt a workup for toxic ingestions. And lastly, a primary hematologic malignancy was also considered, as our patient was in 6th decade of life.

At presentation, her vitals were within normal limits. She was noticeably pale and appeared fatigued. Cardiovascular, respiratory, and abdominal examinations were unremarkable. Musculoskeletal examination was remarkable for mild weakness of bilateral upper extremities against resistance and reduced pinprick sensation in bilateral lower extremities.

## 3 LABORATORY EVALUATION

Urine toxicology, volatiles, acetaminophen, and ethanol panel were all negative. Fecal occult blood test (FOBT) was negative. Anemia studies showed low iron and normal values for ferritin, TIBC, B12, and folate. Reticulocyte count was 2.2%. Peripheral smear showed leukopenia, relative lymphocytosis, and normocytic anemia with anisocytosis and red cell agglutination. Platelet count was within reference range, and no blasts were seen. Bone marrow biopsy was performed, which showed megaloblastoid erythroid hyperplasia with mild dyspoiesis and vacuolization of early erythrocyte precursors—a characteristic feature of copper deficiency. No increased blasts, lymphoid aggregates, or overt malignancy were identified.

**Figure 1:**
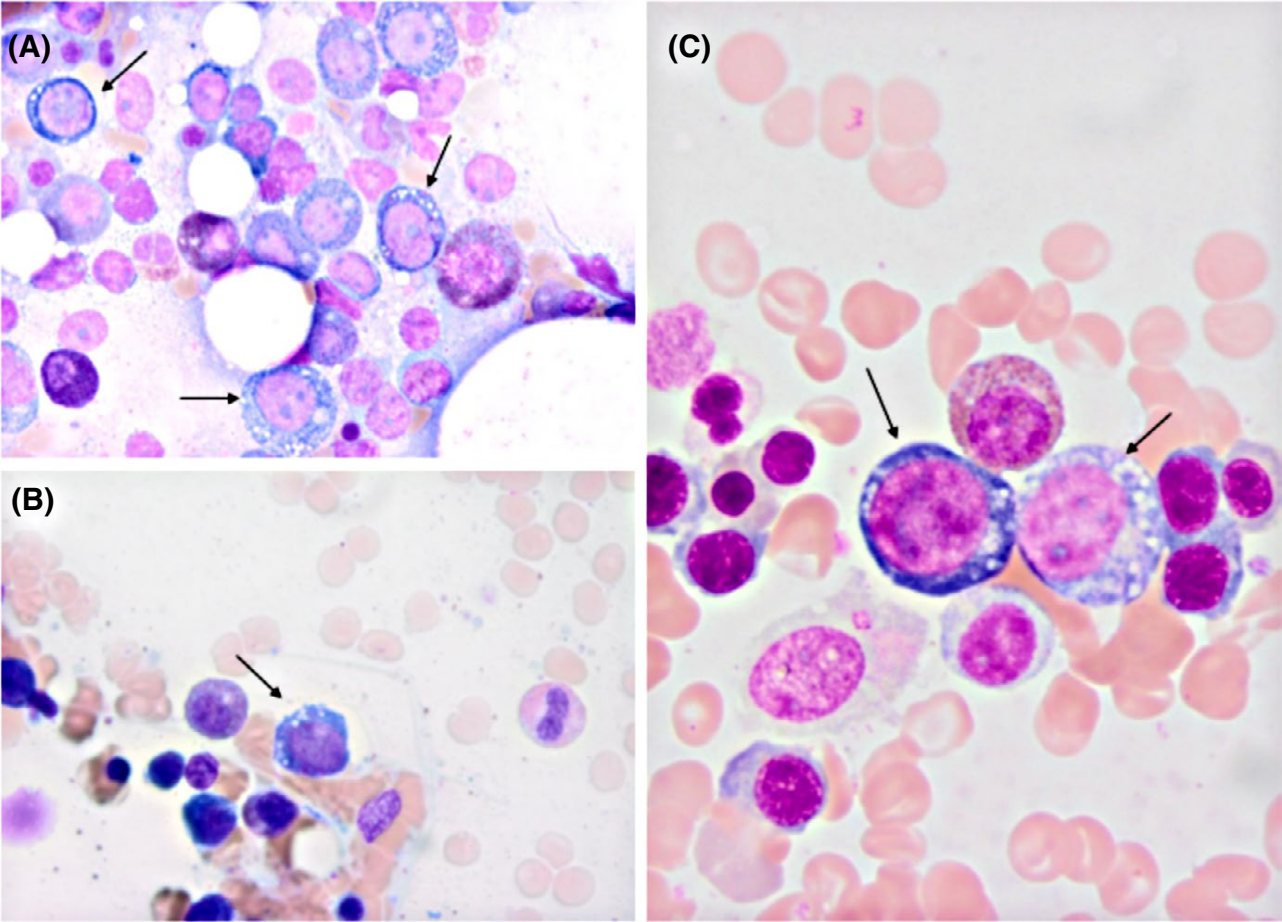
Morphologic changes in marrow precursors in a patient with zinc‐induced copper deficiency. A, Several erythrocyte precursors with multiple white vacuoles within the cytoplasm, a hallmark feature of copper deficiency. However, it is important to know that this finding is nonspecific and can be seen in other conditions, including myelodysplastic syndrome, alcohol intoxication, and drug exposures. B, This photomicrograph shows another erythrocyte precursor with the characteristic vacuolated cytoplasm. C, Two additional erythrocytic precursors with vacuolated cytoplasm

The patient's biopsy specimens are shown in Figure 1. However, it is important to know that this finding is nonspecific and can be seen in other conditions, including myelodysplastic syndrome, alcohol intoxication, and drug exposures.

## 4 DIAGNOSIS AND TREATMENT

A negative FOBT lessened our concern for a gastrointestinal bleed. The gastroenterology service was consulted, but further endoscopic work up was not pursued due to the patient being neutropenic. Nevertheless, malabsorption due to gastric bypass remained a strong possibility, and a multifactorial etiology was considered as the bone marrow biopsy, and clinical history was consistent of bi‐lineage cytopenia secondary to copper deficiency from the excessive zinc ingestion. There was no evidence of malignancy, and alcohol, substance abuse, and medications were excluded as possible explanations. The patient was hemodynamically stable and received a total of three units of packed red blood cells while admitted in the hospital. Though a serum zinc level was never collected as further evidence, the patient was told to discontinue zinc and discharged with copper supplementation under the diagnosis of zinc‐induced hypocupremia. Serum ceruloplasmin and copper were sent out to an outside laboratory, and results both returned after the patient was discharged with levels that were undetectable.

In patients with whom excess zinc ingestion is a cause of copper deficiency, stopping zinc supplementation may suffice and resolve clinical symptoms. However, additional copper supplementation may be required and is generally started initially in addition to discontinuing zinc supplementation. The standard dose for copper supplementation is 2 mg/d. In patients who require higher doses of supplementation, such as our case patient, 8 mg/d can be given for a week, followed by a weekly taper of 2 mg/wk. This regimen was initiated in our patient. Periodic laboratory follow‐up is recommended to determine adequacy of replacement and to assess whether long‐term supplementation is needed.

## 5 FOLLOW‐UP

The patient completed 8 weeks of copper supplementation and presented to the hematology office for follow‐up. She was taking 2 mg daily of copper supplementation, but continued to have fatigue. Laboratory work showed persistent neutropenia and anemia (Hgb 9.6 g/dL, ANC 400 cells/μL). Daily supplementation was increased to 2 mg twice a day. At the 16‐week follow‐up, patient was found to have worsening neutropenia and anemia (Hgb 6.2 g/dL, ANC 100 cells/μL). Copper level continues to be undetectable. Weekly IV copper infusion was initiated for 4 weeks. Three months after initiation of IV infusions, patient's laboratory values have returned to normal (Hgb 12.0 g/dL, ANC 3800 cells/μL) and patient's symptoms have resolved.

The main source of this patient's hypocupremia induced anemia is most likely malabsorption from the duodenum due to gastric bypass surgery, though zinc excess likely contributed to the severity of her clinical course. Copper deficiency in patients with malabsorptive conditions can be even more difficult to correct and may require intravenous replacement if copper levels become critically low. For patients with severe deficiency requiring IV supplementation, 2‐4 mg for 6 days or until asymptomatic is recommended.^7^ Patients with severe deficiency should be aware that despite adequate therapy, hematologic abnormalities may persist 2 months following initiation of treatment and neurologic symptoms may persist longer.

## 6 DISCUSSION

### 6.1 Normal copper metabolism

Dietary copper is absorbed in the stomach as well as the proximal duodenum through a transmembrane protein that transports copper across cell membranes. It is an essential cofactor necessary for maintaining normal nervous system functioning and production of red and white blood cells. The gene ATP7A encodes the copper transmembrane protein transporter, and mutations in this gene lead to impaired copper absorption from gastrointestinal sources and are responsible for the genetic syndrome (Menke's Disease). Secondary deficiencies are relatively rare but are seen in patients with excessive zinc supplementation, use of parenteral nutrition or chronic feeding tube, and various malabsorptive syndromes—including but not limited to gastric bypass, inflammatory bowel disease,^8^ and celiac disease. Severe deficiencies are associated with symptoms such as ataxia,^4^ anemia, neutropenia, polyneuropathy, fatigue, poor wound healing, hair loss, myelopathy, arthralgias, and myalgias. Diagnosis is made simply through copper level measurement. Ceruloplasmin is nonspecific and can be an acute phase reactant.^9^

Copper is involved in cell oxidation and multiple signaling systems. Inhibition of these signaling systems leads to microcytic hypochromic anemia, neutropenia, and peripheral neuropathy. The full mechanisms causing anemia are elaborate, but a few known affected enzyme pathways include a decrease in ceruloplasmin, hephaestin, and cytochrome oxidase. Ceruloplasmin is a copper‐carrying protein, which oxidizes ferrous iron to ferric form and thus allows iron to be transported in circulation and bind to transferrin. Hephaestin, a copper‐dependent transport protein, is involved in iron absorption from erythrocytes, and cytochrome oxidase, a copper‐containing enzyme, contributes to heme synthesis. Copper is an important cofactor in the enzymatic process involved in cell division and protein synthesis leading to neutropenia.^8^ Though simple deficiencies in copper do not cause peripheral neuropathy, severe copper deficiency that are seen more often in zinc excess leads to axonal degeneration and demyelination.^8^ Copper plays a critical role in the structure and function of the central nervous system as it is a part of multiple coenzymes including cytochrome c oxidase, used in mitochondrial electron transport chain; superoxide dismutase, used in oxidative protection; lysyl oxidase, used in cross‐linking collagen and elastin; dopamine Beta‐hydroxylase, used in catecholamine biosynthesis; and peptidylglycine alpha‐amidating mono‐oxidase, used in peptide neurotransmitters hormone processing.^8^

### 6.2 Normal zinc metabolism

Zinc is essential for physiological functions and is involved in multiple biochemical mechanisms contributing to growth and development.^10^ Zinc levels maintain homeostasis via two groups of zinc transporter proteins, ZIPs which shift zinc into the cytosol and ZnTs which shift zinc from the cytosol to extracellular and intracellular compartments.^11^ These transporter proteins help in preventing zinc toxicity; however, there is little research on how gastric bypass surgery and other malabsorptive conditions affect transporter protein expression. Multiple enzymatic processes use zinc as a catalyst and structural component, and in healthy young individuals, Kilic et al found that physiological zinc supplementation increased erythrocytes and leukocytes.^12^ However, in patients with underlying intestinal malabsorption, it is possible that preexisting copper deficiencies are heightened by excessive zinc, outweighing the hematological benefits that may be seen with zinc supplementation.

### 6.3 Copper deficiency with bariatric surgery

Nutritional deficiencies can arise from several mechanisms which include preoperative deficiency (obesity itself is a risk factor), decreased oral intake, chronic malabsorption, and inadequate dietary supplementation.^5^ The bioavailability of copper depends on gastric acid environment. Over 1.3 million people have undergone bariatric surgery between 2011 and 2017, with numbers increasing every year. Around thirty percent are gastric bypasses or revisions.^13^ Sixty‐eight percent of women seeking gastric bypass are deficient in copper prior to bariatric surgery. Ninety percent of patients after biliopancreatic diversion with duodenal switch and 20% of patients after a Roux‐en‐Y were found to be copper deficient. Recommended postoperative nutritional supplements following Roux‐en‐Y bypass surgery include copper, as well as vitamins A, D, E, K, B1, B12, folate, iron, zinc, selenium, and calcium.^14^ Additional goal includes maintaining a ratio of 8‐15 mg of zinc per 1 mg of copper.^7^ In patients who are asymptomatic and those who are receiving adequate nutritional supplements, the cost of routine monitoring may not be justified. However, in patients such as ours who have not been on oral supplementation and are not responding to oral supplementation, sequential laboratory monitoring is necessary, and IV supplementation required.

### 6.4 Copper deficiency with excessive zinc intake

**Figure 2:**
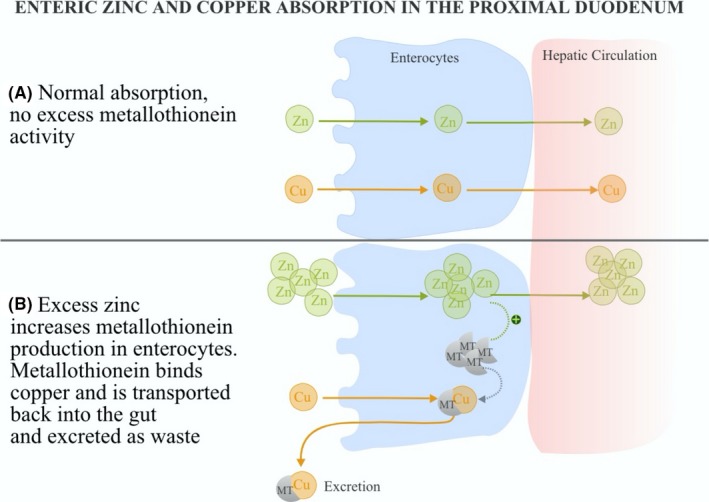
(A) Normal and (B) hypocupremia secondary to zinc supplementation and metallothionein overexpression

Zinc, like copper, is a heavy metal absorbed in the stomach and proximal duodenum. Excessive zinc stimulates enterocytes to produce a heavy metal‐binding protein called metallothionein. Metallothionein is an intracellular protein which reacts rapidly to zinc levels, and high levels of zinc lead to increased metallothionein production. The purpose of this process is to enable metallothionein to bind excess zinc to allow for excretion. However, copper also has a high affinity toward binding to metallothionein and will be sequestered and eliminated if zinc and metallothionein levels are high. A history of zinc supplementation, followed by bi‐cytopenia and neuropathic symptoms, are all clues that lead one to believe that zinc excess is a contributing factor to copper deficiency. Typically, copper deficiency due to excessive zinc resolves with the removal of zinc; however, there are a few cases of persistent hyperzincemia without identifiable external sources. These cases seem to theorize in a primary metabolic defect.^15^ Our case patient is at risk for nutritional deficiency secondary to gastric bypass without receiving adequate supplementation. The increased intake of zinc as promoted by the syndicated TV show further exacerbated her nutritional deficiency in copper, leading to both severe laboratory and clinical symptoms. Schematic of normal and hypocupremia secondary to zinc supplementation and metallothionein overexpression is presented in Figure 2.

### 6.5 Recommended therapy

A summary of recommended therapy and deficiency prophylaxis in patients following gastric bypass is presented in Table 1. Though copper deficiency is rare, it is treatable, so recognition is important. After treatment, hematological derangements normalize in one to three months, and reticulocyte response is followed by hemoglobin recovery.^15^ Although oral copper in patients with intact gastrointestinal tract can correct levels, treatment with IV copper chloride, which in general has no adverse reactions, may be required in patients with impaired gastrointestinal tracts.^16^ Though IV copper repletion results in resolution of hematologic abnormalities, only partial resolution of neurologic deficits was seen in several cases.^5^

**Table 1:** Copper nutritional management regimens

Recommended daily allowance	900 mcg from dietary sources
Maintenance supplementation after biliopancreatic diversion with duodenal switch or Roux‐en‐Y gastric bypass	2 mg PO supplements daily. Maintain a ratio of 8‐15 mg of zinc per 1 mg of copper
Maintenance supplementation after sleeve gastrectomy or gastric banding	1 mg PO supplements daily. Maintain a ratio of 8‐15 mg of zinc per 1 mg of copper
Mild‐to‐moderate deficiency	2‐8 mg PO supplements daily, until levels normalize
Severe deficiency	2‐4 mg intravenous copper for 6 days or until hematologic symptoms resolve

## 7 CONCLUSION

Vitamin and mineral deficiencies are well known to occur in patients with malabsorptive conditions such as gastric bypass. Such underlying deficiencies can be intensified by secondary disorders. Excessive exogenous zinc intake can lead to copper deficiency, causing a specific bi‐lineage deficiency of leukocytes and erythrocytes, with preserved platelet count. Dietary supplementation is common in the outpatient population and must be considered as a possible source of hematologic pathologies.

## CONFLICT OF INTEREST

None declared.

## AUTHOR CONTRIBUTIONS

VT: wrote manuscript and involved in critical revisions of manuscript. JA: conceived idea, assisted in writing manuscript, and involved in patient care. ML: helped identify appropriate images. SM: is primary investigator, involved in patient care, supervised, and reviewed manuscript. All authors read and approved the final version of the manuscript.
